# 基于非靶向代谢组学的缺血性脑卒中生物标志物的分析

**DOI:** 10.3724/SP.J.1123.2024.02015

**Published:** 2025-02-08

**Authors:** Fei XU, Tianping LIU, Yajin GUAN, Weichao HAO, Dingsheng WEN, Shuilin XIE, Yanan BIE

**Affiliations:** 1.广东药科大学基础医学院,广东广州510006; 1. School of Basic Medical Sciences, Guangdong Pharmaceutical University, Guangzhou 510006, China; 2.华南理工大学生物科学与工程学院,广东广州510006; 2. School of Biology and Biological Engineering, South China University of Technology, Guangzhou 510006, China; 3.广东明珠生物技术有限公司,广东佛山528500; 3. Guangdong Mingzhu Biotechnology Co., Ltd., Foshan 528500, China; 4.广东药科大学附属第一医院肿瘤科, 广东广州510062; 4. Department of Oncology, The First Affiliated Hospital of Guangdong Pharmaceutical University, Guangzhou 510062, China; 5.广东药科大学药学院,广东广州510006; 5. College of Pharmacy, Guangdong Pharmaceutical University, Guangzhou 510006, China

**Keywords:** 缺血性脑卒中, 非靶向代谢组学, 生物标志物, 血浆, 脑组织, ischemic stroke (IS), non-target metabolomics, biomarkers, plasma, brain tissue

## Abstract

缺血性脑卒中(ischemic stroke, IS)生物标志物的探索仍未满足临床需求。本研究应用非靶向代谢组学方法,探索IS后血浆和脑组织中的差异代谢物和代谢通路,寻找新的潜在生物标志物和治疗靶点。将12头西藏小型猪随机分为模型组和假手术组,采用额颞入路电凝闭塞大脑中动脉构建缺血性脑卒中模型。术后36 h收集血浆及脑组织样本,采用液相色谱-质谱联用技术进行检测,在*p*<0.05的条件下进行主成分分析与偏最小二乘判别分析,筛选差异代谢物并剔除外源性代谢物,最后进行HMDB(Human Metabolome Database)化合物分类、KEGG通路分析及VIP分析。血浆代谢组学结果显示,86个差异代谢物上调,149个下调,显著差异代谢产物包括(*Z*)-3-氧代-2-(2-戊烯基)-1-环戊乙酸、反式肉桂酸和肉桂酰甘氨酸。脑组织代谢组学结果显示58个差异代谢物上调,53个下调,显著差异代谢物包括2,3-二氢黄酮-3-醇、胍基乙酸(GAA)、*N*-乙酰-D-色氨酸、氧化型谷胱甘肽、2-羟基喹啉和*N*-乙酰-L-天冬氨酸(NAA)。在血浆和脑组织中,前五类化合物中共同化合物类别为有机酸及其衍生物、脂质和脂质样分子、有机杂环化合物以及有机氧化合物。血浆和脑组织的共同代谢通路涉及氨基酸代谢、消化系统、癌症概述和脂类代谢通路。(*Z*)-3-氧代-2-(2-戊烯基)-1-环戊乙酸、GAA、氧化型谷胱甘肽及NAA可能是潜在的生物标志物。本研究为IS的早期筛查和临床治疗方法的开发提供了理论基础。

## Abstract (en)

Biomarkers for ischemic stroke (IS) are yet to fulfill clinical requirements. This study used non-targeted metabolomics to investigate differential metabolites and metabolic pathways in plasma and brain tissue following IS, with the aim of identifying new potential biomarkers and therapeutic targets. Twelve Tibetan miniature pigs were randomly assigned to a model- or sham-operation group. An electrocoagulation-based anterior temporal approach was employed to occlude the middle cerebral artery, thereby creating a model for IS. Plasma and brain tissue samples were collected 36 h post-surgery and analyzed using liquid chromatography-mass spectrometry. Principal component and partial least squares discriminant analyses were used to screen for differential metabolites and exclude exogenous metabolites at *p*<0.05. Compounds were classified according to the HMDB (Human Metabolome Database), and subjected to KEGG (Kyoto Encyclopedia of Genes and Genomes) pathway and VIP (variable importance in projection) analyses. Plasma metabolomics revealed that 86 metabolites were upregulated while 149 were downregulated, with (*Z*)-3-oxo-2-(2-pentenyl)-1-cyclopentylacetic acid, *trans*-cinnamic acid and cinnamoylglycine determined to be significant metabolites. Fifty-eight differential metabolites were upregulated in brain tissue and 53 were downregulated, with 2,3-dihydroflavon-3-ol, guanidinoacetic acid (GAA), *N*-acetyl-D-tryptophan, oxidized glutathione, 2-hydroxyquinoline, and *N*-acetyl-L-aspartate (NAA) identified as significant metabolites. Organic acids and derivatives, lipids and lipid-like molecules, organoheterocyclic compounds, and organic oxygen compounds were found to be common compound categories among the top five types of compound in both plasma and brain tissue. Common metabolic pathways in plasma and brain tissue include amino acid metabolism, digestive system, cancer overview, and lipid metabolism pathways, with the (*Z*)-3-oxo-2-(2-pentenyl)-1-cyclopentylacetic acid, GAA, oxidized glutathione, and NAA metabolites serving as potential biomarkers. This study provides a theoretical foundation for the early screening and development of clinical treatment strategies for IS.

脑卒中是脑血液循环障碍引起的急性神经功能障碍,包括缺血性脑卒中(ischemic stroke, IS)和出血性脑卒中^[1]^。其中,IS是临床卒中中最主要的形式,具有高发病率、高复发率、高流行性、高致残率和高致死率等特点^[2]^。我国是缺血性脑卒中高发国家之一,发病率呈逐年上升趋势^[3,4]^。目前,IS最有效的治疗方法是血管再通治疗,包括静脉溶栓和机械取栓,其成功率与发病时间密切相关^[5,6]^。由于严格的时间窗限制(3~4.5 h),溶栓治疗的效果并不令人满意^[7]^。因此,探索早期筛查的血液和组织生物标志物对IS的治疗具有重要意义。

大脑中动脉(middle cerebral artery, MCA)主要负责大脑半球背外侧面的额叶、颞叶、顶叶、岛叶皮质和内囊膝部、尾状核、豆状核等基底节区的大部分血液供应,是人脑卒中的多发部位。关于大脑中动脉闭塞(middle cerebral artery occlusion, MCAO)动物模型的研究,在啮齿类动物、非人类灵长类动物、犬、兔、猪中均有报道。啮齿类动物因价格低廉、繁殖周期短以及模型制作简单而广泛应用于IS动物模型研究;然而,由于其解剖结构、白质与灰质和人类差异较大,且影像学识别效果差,因此并不是理想的动物模型。相比之下,猪脑的解剖结构、生理生化功能、灰白质分布及比例、形态学发展和神经系统发育与人脑相似。此外,猪对手术的耐受性及抗感染能力更高,猪脑体积更适合进行诱发电位记录、神经外科手术操作以及影像学检测^[8,9]^。因此,本实验采用额颞入路电凝大脑中动脉的方法,成功构建了IS猪模型,术中截断部位恒定,重复性好,缺血效果可靠。

与转录组学和蛋白质组学相比,代谢组学具有高通量、全自动且相对低成本的特点^[10]^,非靶向代谢组学指对生物样本中检测到的所有代谢产物进行全面、系统、无偏向性和无假设的分析,广泛应用于标志代谢物的识别及潜在作用机制的探索。因此,本研究采用非靶向代谢组学筛选猪IS模型中的血浆和脑组织中差异代谢产物,为IS的早期筛查提供潜在的生物标志物,为临床治疗方法的开发提供理论基础。

## 1 实验部分

### 1.1 仪器、试剂与材料

#### 1.1.1 仪器与试剂

Q Exactive HF-X质谱仪、Vanquish Horizon系统超高效液相色谱仪,均购于Thermo Scientific公司;Wonbio-96c多样品冷冻研磨仪(上海万柏生物科技有限公司); JXDC-20氮气吹扫仪(上海净信实业发展有限公司); Centrifuge 5430R高速冷冻离心机(Eppendorf公司); LNG-T88型台式快速离心浓缩干燥器(太仓市华美生化仪器厂)。

色谱纯水、甲醇、异丙醇、乙腈、甲酸,均购于Fisher公司;乙酸铵(色谱纯,Sigma-Aldrich公司)。

#### 1.1.2 实验动物

西藏小型猪,4个月,体重15 kg左右,雄性,普通级,12头,购于广东明珠生物技术有限公司(实验动物生产许可证号:SCXK(粤)2022-0061),饲养于广州华腾生物医药科技有限公司(实验动物使用许可证号:SYXK(粤)2023-0307)。动物实验严格按照国家卫生研究院实验动物护理和使用指南进行,并获得广州华腾生物医药科技有限公司实验动物福利伦理委员会审核及批准,审批编号为HTSW220202,严格遵守3R原则(reduction, replacement, refinement)。

### 1.2 动物实验与样品处理^[11]^

将12头实验动物随机分为两组,一组为大脑中动脉闭塞组(MCAO),一组为假手术组(Sham)。术后36 h收集血浆和脑组织样本。

#### 1.2.1 手术及术后护理

使用2 mg/kg速眠新Ⅱ和3 mg/kg舒泰50肌肉注射麻醉。手术区域消毒后采用额颞入路,在纹路动脉起源近端1.5 mm处找到MCA,电凝诱导缺血。术后肌肉注射150万单位的青链霉素预防感染,氯胺酮(10 mg/kg)给药1 h,静脉注射甘露醇预防水肿。

#### 1.2.2 样品处理

血浆样品处理:移取100 μL猪血浆样品至离心管中,加入400 μL 50%(v/v)甲醇水提取液,涡旋30 s, 5 ℃、40 kHz超声提取30 min。-20 ℃静置30 min后,4 ℃、13000 g离心15 min,取上清液用氮吹仪吹干。加入120 μL 50%(v/v)乙腈水溶液复溶,涡旋后于5 ℃下以40 kHz超声萃取5 min。4 ℃、13000 g离心10 min,取上清液至进样瓶中进行分析。

脑组织样品处理:准确称取猪脑组织100 mg置于研磨管中,加入400 μL 80%(v/v)甲醇水提取液,-10 ℃、50 Hz研磨6 min。5 ℃、40 kHz超声提取30 min。-20 ℃静置30 min后,4 ℃、13000 g离心15 min,取上清液至质谱专用的进样瓶中进行分析。

血浆样品和脑组织样品均需单独设置质量控制(quality control, QC)样本,以评估整个检测过程的稳定性。每个待测样本各取20 μL混合作为QC样本。

#### 1.2.3 苏木精-伊红染色(hematoxylin-eosin staining, HE)

收集脑组织于10%甲醛中固定,经酒精脱水、石蜡包埋、连续切片后按常规步骤进行HE染色。

### 1.3 分析条件

#### 1.3.1 色谱条件

ACQUITY UPLC HSS T3色谱柱(100 mm×2.1 mm, 1.8 μm);柱温:40 ℃;流速:0.4 mL/min。流动相A: 0.1%甲酸水溶液-乙腈(95∶5, v/v);流动相B:含0.1%甲酸的乙腈-异丙醇-水(47.5∶47.5∶5, v/v/v)。流动相洗脱梯度如下:①正离子模式:0~3.0 min, 0B; 3.0~3.1 min, 0B~20%B;3.1~4.5 min, 20%B~35%B; 4.5~5.0 min, 35%B~100%B; 5.0~6.3 min, 100%B; 6.3~6.4 min, 100%B~0B; 6.4~8.0 min, 0B。②负离子模式:0~1.5 min, 0B~5%B; 1.5~2.0 min, 5%B~10%B; 2.0~4.5 min, 10%B~30%B; 4.5~5.0 min, 30%B~100%B; 5.0~6.3 min, 100%B; 6.3~6.4 min, 100%B~0B; 6.4~8.0 min, 0B。

#### 1.3.2 质谱条件

样品经电喷雾电离,分别采用正、负离子扫描模式采集质谱信号;质量扫描范围为*m/z* 70~1050,碰撞能量为20、40、60 eV,鞘气流速为50 arb,辅助气流速为13 arb,喷雾电压为3.5 kV,毛细管温度为325 ℃。

### 1.4 统计学方法

#### 1.4.1 数据预处理

对原始数据缺失值过滤、补值、数据归一化、QC验证和数据转换,以消除或减少实验和分析过程中带来的误差。

#### 1.4.2 代谢产物鉴定

原始数据导入代谢组学处理软件ProgenesisQⅠ(美国Waters公司)进行基线过滤、峰识别、积分、保留时间校正、峰对齐等操作后得到数据矩阵。而后将MS和MS/MS质谱信息与HMDB(http://www.hmdb.ca/)等主流代谢公共数据库以及美吉自建数据库(https://analysis.majorbio.com/)进行匹配,得到代谢物信息。

#### 1.4.3 差异代谢物分析

利用R软件包ropls(Version 1.6.2)对脑组织和血浆中的差异代谢物进行主成分分析(principal component analysis, PCA)、偏最小二乘判别分析(partial least squares discriminant analysis, PLS-DA)和正交最小偏二乘判别分析(orthogonal partial least squares discriminant analysis, OPLS-DA),评估IS模型的稳定性。采用student’s *t*检验和差异倍数分析法,基于OPLS-DA模型的变量权重值VIP > 1和*p*<0.05来确定差异代谢物。

#### 1.4.4 KEGG通路分析

使用Python的统计函数库scipy.stats (Version 1.0.0)进行差异代谢物与通路之间的富集分析,并通过Fisher精确检验得出*p*值,筛选出与MCAO模型相关的差异代谢通路。

实验数据采用IBM SPSS 22进行统计分析。对正态分布或近似正态分布的计量资料用平均值±标准差(mean±SD)进行统计描述。统计采用student’s *t*检验和Tukey的多重比较检验,*p*<0.05被认为具有统计学意义。

## 2 结果与讨论

### 2.1 缺血性脑卒中西藏小型猪模型的建立和验证

本实验采用电凝闭塞右侧大脑中动脉方法构建MCAO模型。两组的大体观察结果及右脑的HE染色结果显示,梗死部位集中在右侧大脑半球的外侧面、岛叶和尾状核,且梗死部位与临床MCA供血区域基本吻合(见图1a、1b)。MCAO猪表现出左前肢无力、蜷曲内缩,原地打圈,后肢无力坐倒,符合临床患者的运动障碍表现,表明本模型已成功建立。

**图 1:**
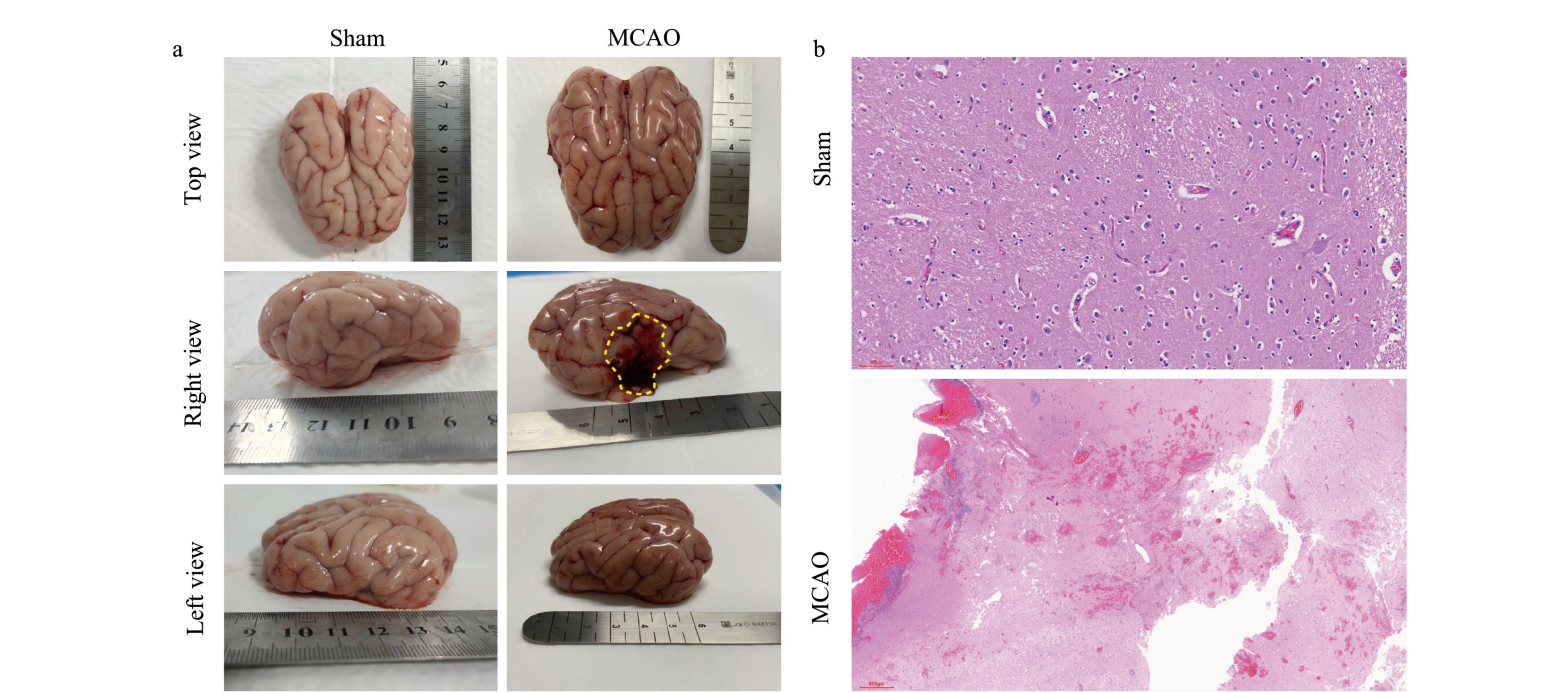
MCAO与Sham组猪脑大体观察与右脑HE染色图

### 2.2 正、负离子模式下PCA及PLS-DA分析

血浆和脑组织检测结果显示,与Sham组相比,在正离子模式下,MCAO组符合差异筛选条件的离子峰数量分别为625和412;在负离子模式下,符合差异筛选条件的离子峰数量分别为819和426。血浆PCA与PLS-DA分析结果表明,正、负离子模式下MCAO和Sham组数据重复性较好,分离明显,两组间代谢产物差异显著,聚合度良好(见图2a、2b)。脑组织PLS-DA分析结果显示,正、负模式下两组间代谢产物差异明显,聚合度良好(见图2d)。

**图 2:**
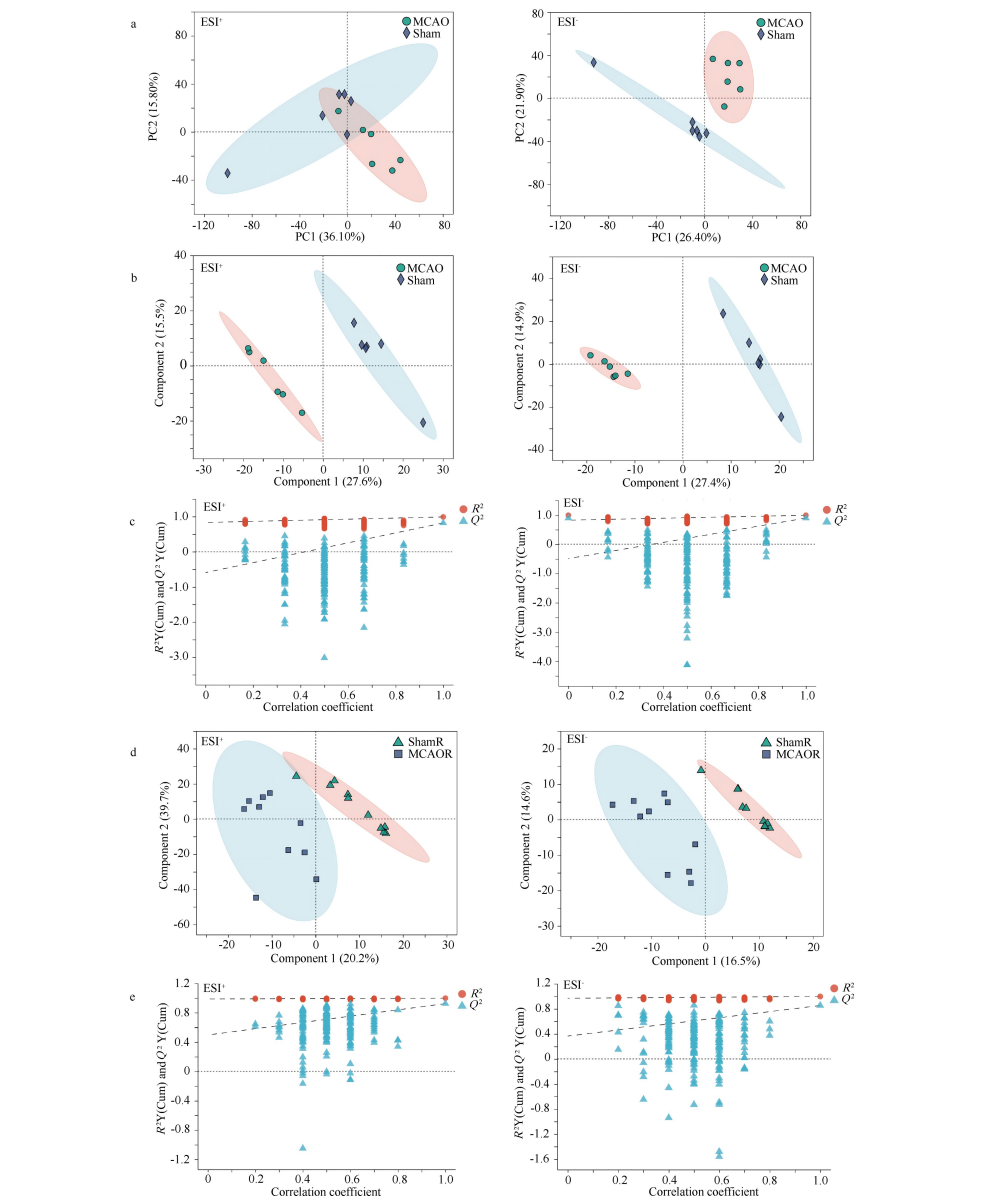
MCAO与Sham组的PCA与PLS-DA分析

在正离子模式下将两个成分进行PLS-DA置换检验。血浆样本中(见图2c)*Q*^2^点的回归线与Y轴的截距均<0.05(正模式下与Y轴截距:*Q*^2^=-0.5925,负模式下与Y轴截距:*Q*^2^=-0.4886),且平均预测能力*Q*^2^均>50%,表明模型稳定可靠、预测能力良好,数据处理后所构建的模式识别模型具有显著统计学意义。脑组织样本中(见图2e)*Q*^2^点的回归线与Y轴的截距>0.05(正模式下与Y轴截距:*Q*^2^=0.4966,负模式下与Y轴截距:*Q*^2^=0.3654),但随着置换保留度的下降,*R*^2^和*Q*^2^置换检验的数值也跟着下降,*Q*^2^点的回归线整体呈向上的趋势,表明置换检验过关,模型不存在过拟合现象,数据处理后所构建的模式识别模型同样具有统计学意义。

### 2.3 差异代谢产物统计

使用OPLS-DA模型得到VIP值,将VIP>1、*p*<0.05的代谢物选为差异代谢物。差异火山图结果显示,正、负离子模式(total模式)下,血浆和脑组织中分别有86和58个上调的差异代谢物,149和53个下调的差异代谢物(见图3)。

**图 3:**
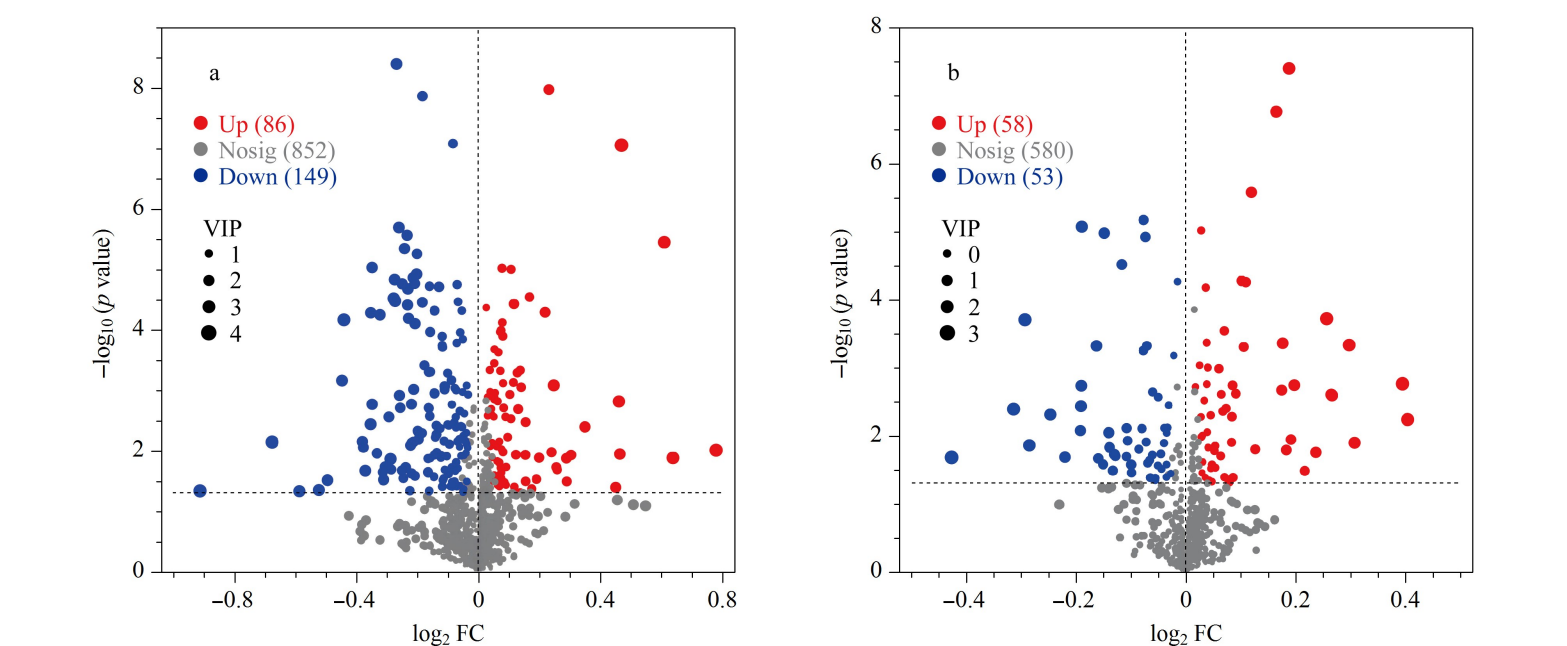
MCAO与Sham组的差异代谢火山图

### 2.4 HMDB化合物分类

对血浆中的代谢物进行HMDB化合物分类分析,结果显示,在正、负离子模式下,代谢物被分为15个类别。其中,TOP 5化合物分类为有机酸和衍生物(29.82%)、脂质和脂质样分子(20.64%)、有机杂环化合物(17.43%)、苯甲酸(10.09%)和有机氧化合物(8.72%)(见图4a)。

**图 4:**
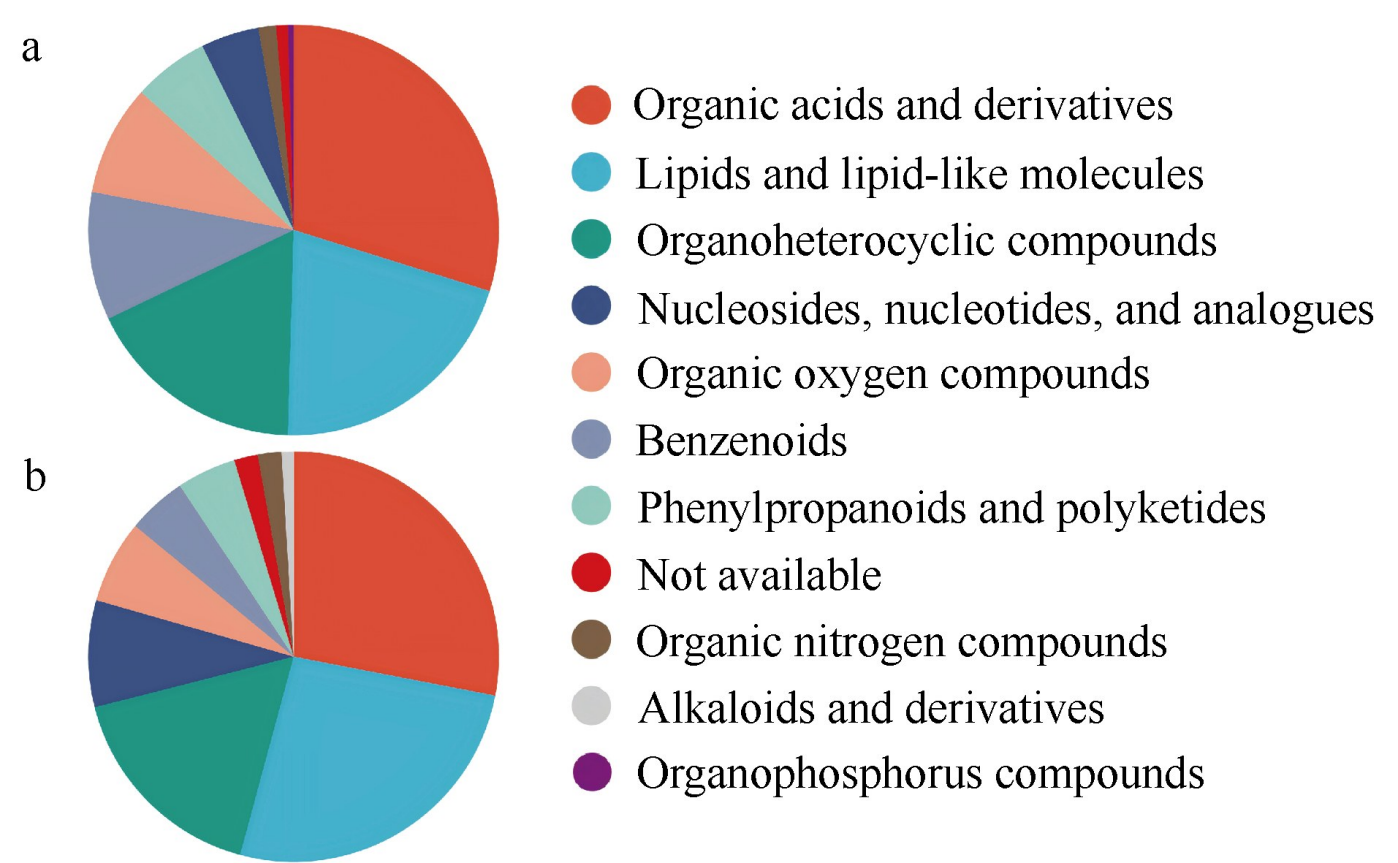
血浆和脑组织的HMDB化合物分类(super class)

脑组织的HMDB化合物分类结果显示,在正、负离子模式下,TOP 5化合物分类为有机酸及其衍生物(28.04%)、脂质和脂质样分子(26.17%)、有机杂环化合物(16.82%)、核苷、核苷酸及其类似物(8.41%)和有机氧化合物(6.54%)(见图4b)。

有机酸及其衍生物、脂质和脂质样分子、有机杂环化合物以及有机氧化合物在血浆和脑组织代谢组学均显示较高的含量。

### 2.5 差异代谢物富集的KEGG通路分析

血浆的KEGG通路结果显示(见图5a),差异代谢物富集的TOP 5功能通路依次为氨基酸代谢(amino acid metabolism)、脂类代谢(lipid metabolism)、癌症概述(cancer: overview)、消化系统(digestive system)和神经系统(nervous system)。脑组织的KEGG通路结果显示(见图5b),差异代谢物富集的TOP 5功能通路依次为氨基酸代谢、脂类代谢、癌症概述、碳水化合物代谢(carbohydrate metabolism)和消化系统。

**图 5:**
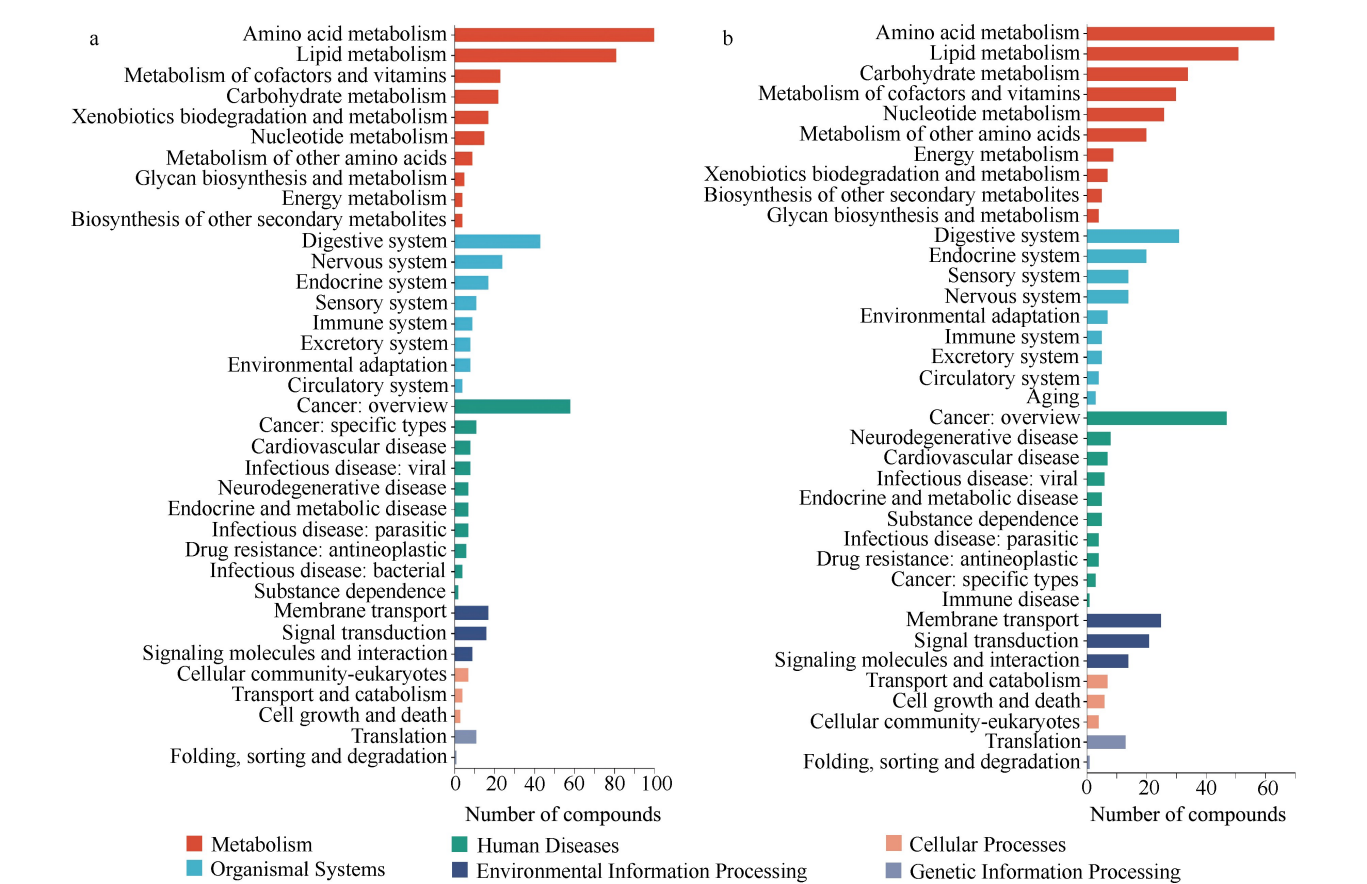
差异代谢物的KEGG功能通路

氨基酸代谢、消化系统、癌症概述和脂类代谢是二者共同代谢通路。IS后血清和脑组织中氨基酸代谢变化显著,可能与某些氨基酸在脑缺血性损伤中的保护作用有关。已有研究表明,赖氨酸和精氨酸能降低脑缺血性损伤的影响^[12]^,谷氨酸也与血栓形成和局部缺血密切相关^[13]^。此外,本研究还提示,IS显著影响一系列与氨基酸代谢、胆碱代谢、脂肪酸代谢、碳水化合物代谢及各种脂质代谢相关的血清代谢物。

### 2.6 差异代谢物的聚类热图和VIP分析

根据差异代谢物的聚类热图和VIP条形图结果,评估IS后血浆和脑组织差异代谢物的重要性和TOP 30代谢物含量的变化趋势。

#### 2.6.1 血浆代谢物聚类热图和VIP分析

图6a结果显示,与Sham组相比,MCAO组中苯丙氨酰组氨酸(phenylalanylhistidine)、(*Z*)-3-氧代-2-(2-戊烯基)-1-环戊乙酸((*Z*)-3-oxo-2-(2-pentenyl)-1-cyclopenteneacetic acid)、*N*-甲基-2-吡咯烷酮(*N*-methyl-2-pyrrolidinone)和(2-乙基巴豆酰基)脲((2-ethylcrotonoyl)urea)等8个代谢物含量显著上升。

**图 6:**
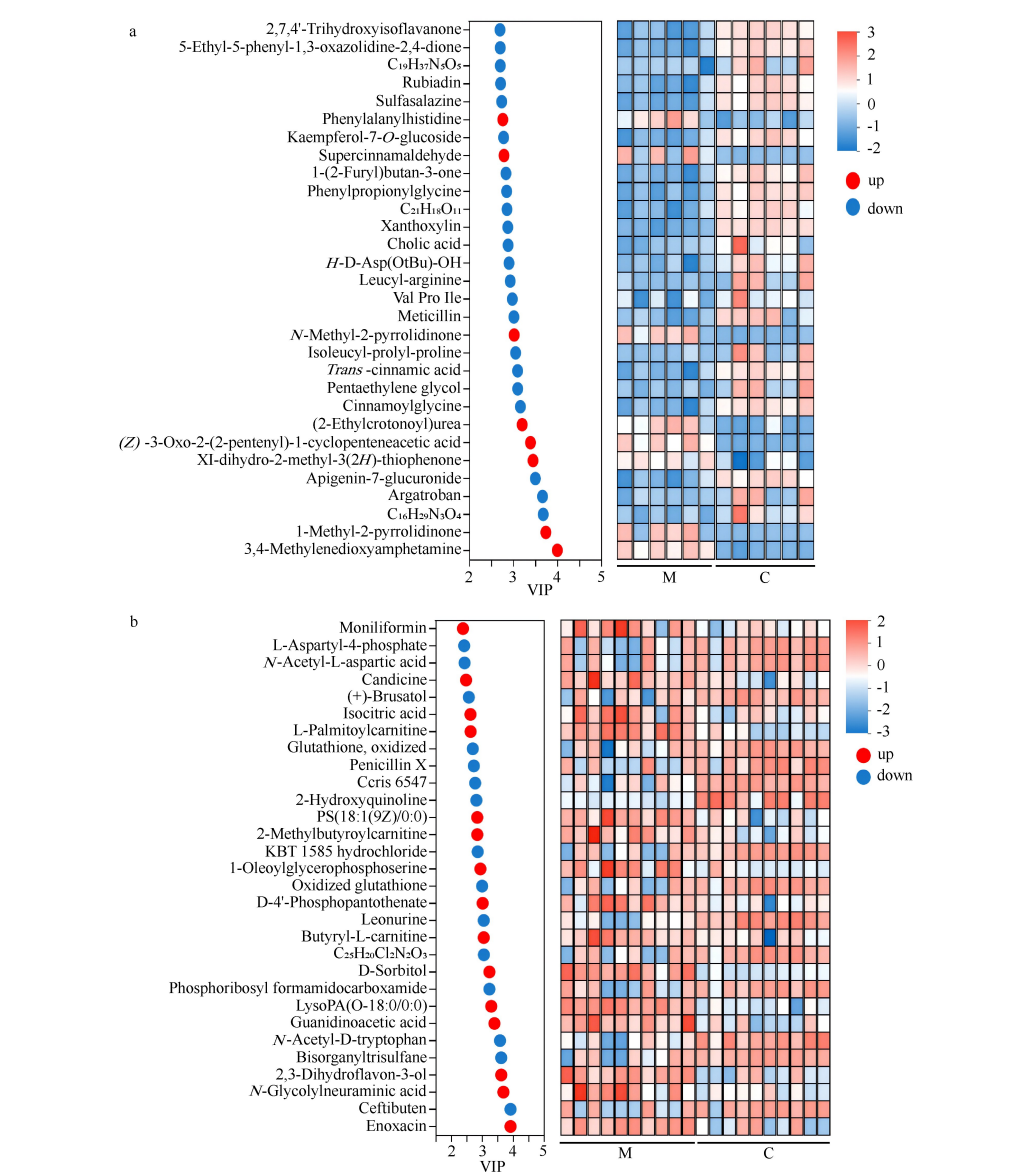
差异代谢物的聚类热图和VIP条形图

MCAO组中D-天冬氨酸-4-叔丁酯(*H*-D-Asp(OtBu)-OH)、反式肉桂酸(*trans*-cinnamic acid)、肉桂酰甘氨酸(cinnamoylglycine)、1-(2-呋喃基)丁-3-酮(1-(2-furyl)butan-3-one)、5-乙基-5-苯基-1,3-恶唑烷-2,4-二酮(5-ethyl-5-phenyl-1,3-oxazolidine-2,4-dione)、黄原氧基(xanthoxylin)、苯丙酰甘氨酸(phenylpropionylglycine)和柳氮磺胺吡啶(sulfasalazine)等22个代谢物含量显著下降。

#### 2.6.2 脑组织代谢物聚类热图和VIP分析

图6b结果显示,与Sham组相比,MCAO组中2,3-二氢黄酮-3-醇(2,3-dihydroflavon-3-ol)、2-甲基丁酰基肉毒碱(2-methylbutyroylcarnitine)、胍基乙酸(guanidinoacetic acid, GAA)、*N*-羟乙酰神经氨酸(*N*-glycolylneuraminic acid)和异柠檬酸(isocitric acid)等15种代谢物的含量显著上升。同时,*N*-乙酰-D-色氨酸(*N*-acetyl-D-tryptophan)、氧化型谷胱甘肽(oxidized glutathione)、2-羟基喹啉(2-hydroxyquinoline)、*N*-乙酰-L-天冬氨酸(*N*-acetyl-L-aspartic acid)和磷酸核糖基甲酰胺(phosphoribosyl formamidocarboxamide)等15种代谢物的含量显著下降。

谷胱甘肽(GSH)是由谷氨酸、半胱氨酸和甘氨酸缩合而成的低相对分子质量含硫假三肽。它在保护蛋白质硫醇基团免受氧化应激损伤和中和活性氧方面发挥重要作用。谷胱甘肽有两种形式:还原型(GSH)和氧化型(GSSG)。在脑组织中,谷胱甘肽主要以高浓度(1~3 mmol/L)的还原型存在。在IS模型中,作为氧化应激标志物的GSH稳态在脑组织和血浆中均受到影响。GSH水平可能为IS期间的氧化应激提供适应机制^[14]^。

胍基乙酸是一种氨基酸衍生物,主要在体内合成,与能量代谢和神经保护机制有关。GAA是肌酸的天然前体,在胍基乙酸*N*-甲基转移酶(GAMT)催化作用下,内源性合成肌酸参与三磷酸腺苷(ATP)循环^[15⇓-17]^。GAA能激活脑和外周组织中的*γ*-氨基丁酸(GABA)受体。研究表明,GAMT缺乏会导致GAA合成肌酸的过程受阻,导致血清或脑组织中累积高浓度的GAA,引发自发性的神经元损伤。这表明,GAA作为GABA竞争性抑制剂,激活可能影响神经系统的兴奋性^[18,19]^。本研究发现,IS后GAA含量升高。因此,我们推测GAMT及GAA可能是IS的潜在生物标志物。

*N*-乙酰-L-天冬氨酸(NAA)是神经元内特有的氨基酸衍生物,由天冬氨酸和乙酰辅酶A合成。它参与神经系统的多种过程,如神经元蛋白合成的调节、髓鞘脂质合成以及多种神经递质(如天冬氨酸或NAA)的代谢^[20⇓-22]^。研究表明,缺血后NAA水平显著下降,与脑组织损伤的程度相关^[23]^。在急性缺血性脑卒中患者中,NAA浓度显著降低,能够反映神经元的存活情况,从而帮助评估缺血的严重程度^[24]^。此外,NAA水平在缺血事件后的早期阶段(如24 h内)能够预测后期的脑梗死发展^[25]^,是一个有价值的预后生物标志物。

## 3 结论

目前,国内外暂无有效的IS生物标志物,因此开发能够准确预测IS的血浆和脑组织生物标志物显得尤为迫切。本研究采用额颞入路电凝大脑中动脉的方法成功构建了猪缺血性脑卒中模型,并应用非靶向代谢组学方法研究IS后脑组织和血浆的共同差异代谢物和代谢通路。我们识别出(*Z*)-3-氧代-2-(2-戊烯基)-1-环戊乙酸、GAA、氧化型谷胱甘肽和NAA等作为潜在生物标志物。这一发现不仅为进一步研究提供了新的方向,还可能为临床早期筛查和治疗策略的制定奠定基础。未来的研究应关注于验证这些生物标志物的临床适用性,并探索其他动物模型的应用,以增强研究结果的广泛性和适用性。
